# The KCa3.1 K^+^ Channel and Cardiovascular Disease: An Upstream Target Linking Inflammation, Fibrosis and Electrical Instability

**DOI:** 10.3390/cells15050416

**Published:** 2026-02-27

**Authors:** Ibrahim Antoun, Georgia R. Layton, Riyaz Somani, G. André Ng, Peter Bradding, Mustafa Zakkar

**Affiliations:** 1 Department of Cardiology, University Hospitals of Leicester NHS Trust, Glenfield Hospital, Leicester LE3 9QP, UK; 2 College of Health Sciences, University of Leicester, Glenfield Hospital, Leicester LE3 9QP, UK; 3 Department of Respiratory Sciences, University of Leicester, Leicester LE1 9QP, UK; 4 Department of Cardiac Surgery, University Hospitals of Leicester NHS Trust, Glenfield Hospital, Leicester LE3 9QP, UK; 5 National Institute for Health Research Leicester Research Biomedical Centre, Leicester LE5 4PW, UK; 6 Leicester British Heart Foundation Centre of Research Excellence, Glenfield Hospital, Groby Road, Leicester LE3 9QP, UK

**Keywords:** KCa3.1, atrial fibrillation, atherosclerosis, cardiac fibrosis

## Abstract

KCa3.1 encodes the intermediate-conductance calcium-activated potassium channel KCa3.1, a regulator of membrane potential and calcium-dependent signalling in cardiovascular and immune cells. Increasing evidence indicates that KCa3.1 is a shared driver of vascular remodelling, inflammation, fibrosis, and electrical instability across multiple cardiovascular diseases. In ischaemic heart disease (IHD), KCa3.1 is upregulated in endothelial cells, vascular smooth muscle cells, macrophages, and T lymphocytes, where it promotes smooth muscle proliferation, neointimal formation, and chronic vascular inflammation. Genetic deletion or pharmacological blockade of KCa3.1 reduces atherosclerotic plaque burden and restenosis in animal models. In atrial fibrillation (AF), KCa3.1 contributes to electrical remodelling by shortening atrial action potential duration and to structural remodelling by driving fibroblast activation and collagen deposition. KCa3.1 also regulates macrophage polarisation and pro-inflammatory cytokine release in atrial tissue, linking immune activation to arrhythmogenic substrate formation. Inhibition of KCa3.1 prolongs atrial refractoriness, attenuates atrial fibrosis, and reduces AF inducibility in multiple preclinical models. Emerging data in valvular heart disease suggest that KCa3.1 is upregulated in valvular interstitial cells and regions of active calcification, where it supports myofibroblast differentiation, osteogenic signalling, and inflammatory crosstalk, implicating the channel in fibrocalcific valve degeneration. Collectively, these findings position KCa3.1 as a central molecular integrator of electrical, fibrotic, and inflammatory pathways in cardiovascular disease. The availability of selective KCa3.1 inhibitors with established human safety profiles supports the feasibility of therapeutic translation. Targeting KCa3.1 may enable disease-modifying strategies that extend beyond symptom control to suppress maladaptive cardiovascular remodelling.

## 1. Introduction

KCa3.1 encodes the intermediate-conductance calcium-activated potassium channel KCa3.1 (also previously known as IK1, IKCa4 and SK4). KCa3.1 channels are widely expressed in both cardiovascular cells (endothelial cells, vascular smooth muscle cells, fibroblasts) and immune cells (T lymphocytes, macrophages) [1]. Activation of KCa3.1 allows K^+^ efflux in response to intracellular Ca^2+^ elevations, hyperpolarising the cell membrane and thereby modulating calcium signalling. Through this mechanism, KCa3.1 critically regulates membrane potential, Ca^2+^ influx, and downstream cellular functions, including proliferation, migration, and secretion. In excitable tissues such as the heart, KCa3.1 channels modulate cardiac electrophysiology by influencing action potential duration and refractoriness in a context-dependent manner, with potential contributions to both physiological repolarisation and pathological arrhythmogenesis [2]. In non-excitable cells, KCa3.1 serves as a key facilitator of Ca^2+^-dependent responses, including cytokine release in immune cells and phenotypic switching in vascular cells.

**Figure 1:**
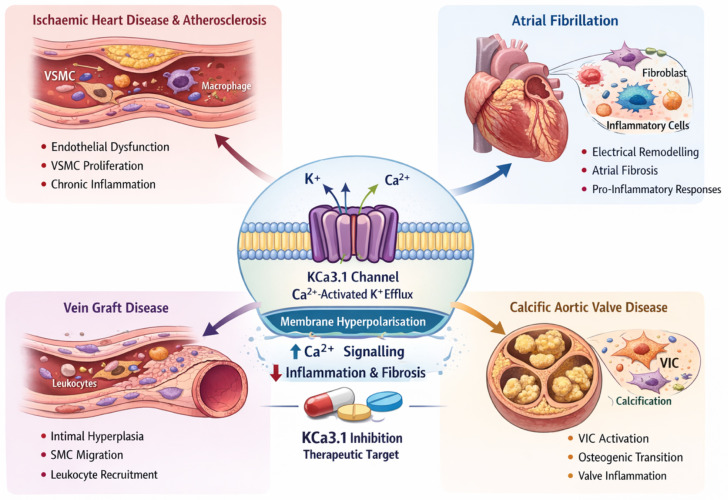
Schematic overview of KCa3.1 channel biology across major cardiovascular phenotypes. The central panel depicts Ca^2+^-activated K^+^ efflux through KCa3.1, leading to membrane hyperpolarisation and amplification of Ca^2+^-dependent signalling in cardiovascular and immune cells. In ischaemic heart disease and atherosclerosis, increased KCa3.1 activity promotes vascular smooth muscle cell phenotypic switching and immune cell activation, leading to local release of pro-inflammatory cytokines within the plaque. In atrial fibrillation, KCa3.1 contributes to electrical remodelling, shortening atrial repolarisation, and supports fibroinflammatory structural remodelling via KCa3.1 expressed on fibroblast membranes and inflammatory cells. In valvular heart disease, KCa3.1 is implicated in valvular interstitial cell activation, osteogenic signalling, and inflammatory crosstalk, contributing to fibrocalcific valve degeneration. Therapeutic targeting highlights selective KCa3.1 inhibition as a disease-modifying strategy with potential to attenuate remodelling, suppress inflammation, and reduce arrhythmia burden. ↑ indicates increased values; ↓ indicates decreased values.

Emerging evidence implicates KCa3.1 in the pathogenesis of major cardiovascular diseases, notably ischaemic heart disease (IHD), atrial fibrillation (AF), and valvular heart disease, by promoting vascular remodelling, inflammation, fibrosis, and arrhythmogenesis [3]. These convergent mechanisms are illustrated schematically in Figure 1. This narrative review summarises the current understanding of KCa3.1’s roles in these conditions, highlighting major studies and potential therapeutic implications. We discuss how KCa3.1-mediated pathways contribute to atherosclerosis in IHD, to the electrical and structural remodelling underlying AF, and to the fibrotic and calcific degeneration of cardiac valves.

KCa3.1, intermediate conductance calcium-activated potassium channel 3.1; K^+^, potassium ion; Ca^2+^, calcium ion; VSMC, vascular smooth muscle cell; VIC, valvular interstitial cell; CAVD, calcific aortic valve disease.

**Signalling pathways downstream of KCa3.1**

KCa3.1 acts as an upstream amplifier of Ca^2+^-dependent signalling rather than a passive ion channel. Channel activation induces membrane hyperpolarisation, which increases the electrochemical gradient for Ca^2+^ entry through store-operated Ca^2+^ channels [4]. Sustained Ca^2+^ influx activates key intracellular pathways, including PI3K/AKT, MAPK/ERK, and NF-κB signalling. In vascular smooth muscle cells, KCa3.1-dependent Ca^2+^ entry promotes phenotypic modulation and enhances proliferative and migratory signalling, including activation of the MAPK/ERK and PI3K/AKT pathways [5]. In macrophages, KCa3.1-dependent Ca^2+^ signalling enhances NF-κB activation and drives the release of pro-inflammatory cytokines, including IL-1β and TNF-α, thereby reinforcing plaque inflammation. In T lymphocytes, KCa3.1 maintains Ca^2+^ oscillations required for calcineurin activation and NFAT translocation, thereby supporting T-cell activation, proliferation, and cytokine production [4]. These converging pathways position KCa3.1 as a central regulator linking ionic flux to transcriptional programmes that drive vascular remodelling and immune-mediated disease progression. An important mechanistic consideration is the differential effect of KCa3.1-mediated membrane hyperpolarisation on distinct Ca^2+^ entry pathways. In non-excitable cells, including endothelial cells, fibroblasts, and immune cells, KCa3.1 enhances Ca^2+^ influx primarily through store-operated Ca^2+^ channels and other non-voltage-gated pathways by increasing the electrochemical driving force for Ca^2+^ entry [6]. In contrast, in cells that express voltage-gated Ca^2+^ channels, membrane hyperpolarisation may reduce CaV channel activation by shifting the membrane potential further from the activation threshold [7]. These apparently opposing effects are not contradictory but reflect cell-type-specific channel expression and functional hierarchy. In proliferative and inflammatory cells, Ca^2+^ entry is largely mediated by store-operated pathways, and KCa3.1 acts as a critical amplifier of sustained Ca^2+^ signalling. In more excitable phenotypes, the balance between suppression of voltage-gated Ca^2+^ influx and facilitation of non-voltage-gated Ca^2+^ entry will determine the net physiological effect [8]. This context-dependent integration of Ca^2+^ signalling pathways is central to understanding how KCa3.1 drives vascular remodelling, fibrosis, and immune activation.

## 2. KCa3.1 in Ischaemic Heart Disease and Vascular Remodelling

IHD is driven largely by atherosclerosis and adverse vascular remodelling. KCa3.1 is now recognised as a pro-atherogenic factor in this context [9]. Expression of KCa3.1 is upregulated in vascular cells and lesional immune cells during atherosclerosis [9]. A previous study showed that human coronary arteries from patients with coronary artery disease have elevated KCa3.1 levels [10]. In the ApoE^(^^−/−)^ mouse model of atherosclerosis, KCa3.1 expression is markedly increased in the vascular smooth muscle cells (VSMCs) of the intima and media, as well as in infiltrating macrophages and T-lymphocytes within plaques [10]. This spatiotemporal upregulation suggests KCa3.1 is engaged in the disease’s key cellular players.

Mechanistically, KCa3.1 promotes the transition of VSMCs to a proliferative, migratory phenotype that contributes to neointimal hyperplasia and fibrous cap formation. In vitro, blocking or silencing KCa3.1 suppresses VSMC proliferation, migration, and reactive oxygen species generation [11]. Concordantly, KCa3.1 knockout mice show reduced VSMC proliferation and lower macrophage activation in vascular injury models. KCa3.1 also regulates endothelial function: in healthy vessels, endothelial KCa2.3/KCa3.1 channels mediate endothelium-dependent hyperpolarisation and vasodilator responses [12]. Moreover, KCa3.1 in endothelial cells and circulating bone marrow-derived cells influences neovessel formation (angiogenesis) in plaques and post-ischaemic tissues [13].

Importantly, KCa3.1 activity in immune cells links it to chronic vascular inflammation. Macrophages and T cells require KCa3.1 for full activation; the channel sustains Ca^2+^ signalling during immune cell activation and cytokine production [14]. In atherosclerotic plaques, KCa3.1 facilitates the pro-inflammatory phenotype of macrophages and T helper cells, thereby exacerbating inflammation and plaque instability. Indeed, genetic deletion of KCa3.1 in ApoE^(−/−)^ mice or pharmacological blockade leads to fewer inflammatory cells in plaques. For example, KCa3.1 blockade reduced macrophage and T-lymphocyte infiltration into aortic plaques, indicating a dampened inflammatory response [15].

The pathogenic contributions of KCa3.1 in IHD are underscored by therapeutic studies in animal models. In ApoE^(−/−)^ mice, in vivo therapy with KCa3.1 blockers (TRAM-34 or clotrimazole) significantly reduced atherosclerotic lesion development. Treated mice showed smaller aortic plaques with decreased VSMC content, reduced macrophage/T-cell infiltration, and reduced oxidative stress. Similarly, local delivery of TRAM-34 to injured coronary arteries in a swine model of angioplasty prevented acute VSMC phenotypic switching and limited neointimal stenosis [16]. These findings indicate that KCa3.1 drives adverse vascular remodelling, and its inhibition can preserve vascular structure and function after injury. Notably, chronic KCa3.1 blockade in mice caused no major toxicity or immunosuppression; for instance, TRAM-34 did not compromise normal immune response to viral infection. This provides proof of concept that KCa3.1 is a safe and effective target for attenuating atherosclerosis and restenosis [17].

Many patients with significant coronary disease (CAD) will require invasive intervention such as percutaneous coronary intervention (PCI) or coronary artery bypass grafting (CABG). Different conduits are currently used during surgery, including the internal mammary artery (IMA) and the long saphenous vein (LSV), which is the most frequently used conduit in CABG by the number of grafts [18]. Although the IMA has excellent long-term patency, its length and availability limit its use, and it is commonly used to graft the left anterior descending artery (LAD). Vein grafts on the other side have lower patency rates due to vein graft disease (VGD), leading to graft failure in up to 50% of patients 10 years post-surgery and limiting the long-term success of CABG. This results in the recurrence of symptoms, increased risk of myocardial infarction and the need for repeat revascularisation, with females being more often affected than men [19]. The development of VGD results from intimal hyperplasia (IH) driven by multifactorial processes. These initiate early in veins through activation of endothelial cells (EC), which, in turn, trigger smooth muscle cell (SMC) proliferation, migration, and leukocyte recruitment [20]. There are no effective prevention strategies for VGD despite years of research. These processes are closely aligned with pathways regulated by KCa3.1. Endothelial activation, leukocyte recruitment, and smooth muscle cell proliferation are all driven by Ca^2+^-dependent signalling, which is sustained by KCa3.1-mediated membrane hyperpolarisation [21]. In this context, KCa3.1 provides a mechanistic link between vascular injury and subsequent remodelling, suggesting that modulation of this channel may influence early graft failure and restenosis.

Very limited evidence exists on the expression, distribution, and vasodilatory contribution of the calcium-activated potassium (KCa) channel family (KCa1.1; KCa2.1, 2.2, 2.3; KCa3.1) in these commonly used coronary artery bypass grafts [22]. Sun et al. showed that both the IMA and LSV express all KCa channel subtypes, with each subtype distributed in both endothelium and smooth muscle. The IMA and LSV did not differ in the overall expression level of each KCa channel subtype, corresponding to comparable relaxant responses to respective subtype activators. In the IMA, BKCa was more abundantly expressed in smooth muscle than in endothelium, whereas SKCa was greater in the endothelium. In comparison, the LSV showed even distribution of KCa channel subtypes in the 2 layers. [23]. This study suggested intrinsic differences between the two grafts that could potentially affect postoperative function, raising the question of the role of KCa3.1 modulation on improving LSV patency, considering that KCa3.1 is known to modulate pathways such as inflammation and mesenchymal transition that are known to play an essential role in the development of vein graft disease [24,25].

KCa3.1 also plays a significant role in percutaneous coronary intervention (PCI) failure, particularly by promoting in-stent restenosis (ISR) through the activation of VSMC proliferation and migration. Increased expression and activity of KCa3.1 is associated with the phenotypic modulation of coronary smooth muscle cells from a contractile to a proliferative state [26,27]. This phenotypic shift is a key driver of neointimal hyperplasia, the primary mechanism of restenosis after stent implantation. KCa3.1 activation causes membrane hyperpolarisation, which increases the driving force for Ca^2+^ influx. This rise in intracellular Ca^2+^ activates mitogenic signalling in VSMCs, including ERK1/2 activation downstream of IKCa- or KCa3.1-dependent Ca^2+^ entry, and PI3K pathway involvement in contexts where KCa3.1 upregulation drives VSMC migration and proliferation [28]. KCa3.1 is also involved in macrophage activation and migration. In the context of vascular injury, KCa3.1-mediated macrophage activation can promote inflammation, further exacerbating the restenotic process. Studies indicate that blocking KCa3.1, for instance, with the inhibitor TRAM-34, can reduce VSMC proliferation and migration [26,29]. Therefore, KCa3.1 is a potential therapeutic target for preventing post-angioplasty restenosis.

Beyond atherosclerosis, KCa3.1 is also implicated in post-myocardial infarction remodelling. Cardiac ischaemia and heart failure are associated with microvascular rarefaction; KCa3.1 upregulation in these settings (as observed in rodent models of myocardial infarction and hypertension) may contribute to impaired vasodilation and increased vascular resistance [30,31]. By modulating fibroblast and immune cell activity, KCa3.1 may also influence infarct healing and scar formation.

KCa3.1 also plays an important physiological role in endothelial homeostasis. Together with KCa2.3 channels, endothelial KCa3.1 contributes to endothelium-dependent hyperpolarisation, a key mechanism underlying vasodilation, particularly in resistance vessels. Activation of these channels promotes membrane hyperpolarisation, which enhances calcium entry and stimulates the release of vasodilatory and antiproliferative mediators, including nitric oxide and endothelium-derived hyperpolarising factor [32]. Through these mechanisms, KCa3.1 supports vascular relaxation, limits smooth muscle cell proliferation, and maintains microvascular function. These physiological roles raise the theoretical concern that pharmacological inhibition of KCa3.1 could impair endothelial-dependent vasodilation or vascular repair. However, available preclinical and early phase clinical data have not demonstrated clinically significant adverse vascular effects with KCa3.1 inhibition, suggesting that partial or context-dependent blockade may preferentially target pathological signalling while preserving essential endothelial function [9]. Further human studies are required to define the balance between therapeutic benefit and potential vascular effects.

## 3. KCa3.1 in AF: Electrophysiology and Fibrosis

AF is increasing in prevalence, and its management is challenging, especially in the developing world [33]. AF is characterised by both electrical remodelling (action potential alterations, ion channel dysregulation) and structural remodelling (atrial fibrosis and dilation). Compelling evidence now links KCa3.1 to both processes in AF, making it a unique contributor to arrhythmogenesis.

1. Electrophysiological Role in Atria: Unlike the ventricle, atrial cardiomyocytes express small- and intermediate-conductance Ca^2+^-activated K^+^ currents that influence repolarisation. Recent studies indicate that KCa3.1 channel activity shortens the atrial action potential duration (APD) under conditions of atrial stress. For instance, a previous study found that, in a cellular AF model (atrial myocytes subjected to rapid pacing), KCa3.1 current density and protein expression were significantly increased, leading to a shortening of APD. This APD shortening creates a vulnerable substrate for re-entry. When the KCa3.1 blocker TRAM-34 was applied to paced atrial myocytes, it prolonged the action potential and reduced the susceptibility to induced AF. Thus, KCa3.1 contributes to atrial repolarisation; its upregulation during AF or rapid pacing can destabilise the atrial rhythm by abbreviating refractoriness [34]. Interestingly, one mechanism for KCa3.1 upregulation in myocytes involves crosstalk with fibroblasts: atrial fibroblast-derived exosomes enriched in miR-21 were shown to increase KCa3.1 expression in atrial myocytes via the PI3K/Akt pathway during rapid pacing. This novel finding links fibrotic signalling to ion channel remodelling, illustrating how fibroblast-myocyte communication can amplify arrhythmogenic KCa3.1 activity.

Beyond direct ion current effects, KCa3.1 may influence Ca^2+^ handling in atrial cells. By hyperpolarising the membrane, KCa3.1 activity may influence intracellular Ca^2+^ handling by increasing the driving force for Ca^2+^ entry through non-voltage-gated pathways and by favouring forward mode Na^+^/Ca^2+^ exchanger activity, which promotes Ca^2+^ extrusion while generating a net inward depolarising current that can contribute to triggered activity under pathological conditions [35]. Overall, the electrical remodelling component of AF involves KCa3.1-driven AP shortening and possibly Ca^2+^ dysregulation, which together favour re-entrant and focal arrhythmias. Targeting KCa3.1 may reverse some electrical abnormalities in AF; however, excessive prolongation of action potential duration may itself be proarrhythmic, underscoring the need for controlled and context-dependent modulation of KCa3.1 activity [36].

2. KCa3.1 and Atrial Fibrosis: Atrial fibrosis is a hallmark of the AF substrate, providing the structural heterogeneity that sustains AF. KCa3.1 is strongly implicated in fibroblast and myofibroblast activation in the atria. In both atrial-specific studies and systemic cardiac fibrosis models, KCa3.1 promoted fibroblast proliferation and collagen secretion [37]. Angiotensin II, a pro-fibrotic stimulus in atria, upregulates KCa3.1 expression in atrial fibroblasts via the ERK and NF-κB signalling pathways. This KCa3.1 induction is critical for the fibrotic response: blocking KCa3.1 with TRAM-34 or silencing with siRNA significantly reduced Ang II-dependent atrial fibroblast proliferation and extracellular matrix (collagen) production. In vivo, chronic Ang II infusion in rats induced atrial fibrosis accompanied by upregulation of KCa3.1 in atrial tissue [38], whereas rats treated with the AT1-receptor blocker losartan or TRAM-34 showed attenuated atrial fibrosis and lower KCa3.1 levels [39]. These findings confirm that KCa3.1 is a driver of atrial fibroblast activation and fibrogenesis, and importantly, that KCa3.1 inhibition can prevent or reverse atrial fibrosis.

KCa3.1’s role in cardiac fibrosis is not limited to resident fibroblasts. A study in a systemic fibrosis model (Ang II-treated rats) demonstrated that KCa3.1 channels create a pro-fibrotic, inflammatory microenvironment in the heart by acting on circulating monocytes and T cells [40]. Gang She et al. showed that KCa3.1 is expressed in ventricular fibroblasts and bone marrow-derived mononuclear cells; Ang II increased KCa3.1 expression in these cells, linking inflammation to fibrosis. Pharmacologic KCa3.1 blockade (TRAM-34) in that model dramatically reduced cardiac fibrosis and inflammation, with fewer myofibroblasts in the myocardium [40]. Notably, TRAM-34 suppressed the release of IL-4 and IL-13 from CD4^+^ T-cells and, in turn IL-4/IL-13-driven differentiation of monocytes into fibroblast-like cells (fibrocytes). These cytokines promote alternative macrophage activation and fibrogenesis. Thus, KCa3.1 links immune signalling to fibroblast recruitment, and blocking KCa3.1 reduced pro-fibrotic Th2 cytokines and prevented monocyte-to-fibroblast differentiation, breaking a key feed-forward loop in fibrosis. Although this study was conducted in the ventricles, a similar immune-fibrotic interplay is likely relevant to atrial fibrosis.

3. KCa3.1 and Atrial Inflammation: Inflammation in atrial tissue is increasingly recognised as a contributor to AF maintenance, especially in secondary AF (e.g., post-cardiac surgery, heart failure-associated AF). Macrophages infiltrating the atrial myocardium release cytokines that affect conduction and promote fibrosis. Recent research highlights KCa3.1 as a key regulator of macrophage inflammatory polarisation in the atrium [40]. In a canine model of AF induced by prolonged atrial tachy-pacing, KCa3.1 expression was upregulated in atrial macrophages, correlating with a shift toward pro-inflammatory M1 polarisation. He et al. demonstrated that administering TRAM-34 to these dogs markedly altered the atrial inflammatory profile [40]. KCa3.1 blockade attenuated macrophage M1 polarisation in the atrium and significantly reduced atrial levels of IL-1β, TNF-α, and CC-chemokine ligand 2 (CCL2). This was accompanied by a decrease in AF inducibility and duration in treated animals [41]. Essentially, inhibiting KCa3.1 quelled the local inflammatory response, thereby reducing the arrhythmogenic substrate and suppressing AF. The study further suggested that the p38 MAPK/AP-1/NF-κB pathways were involved in KCa3.1-driven macrophage polarisation, as TRAM-34’s effects mirrored what p38 inhibition would achieve (shifting macrophages toward an M2 phenotype, characterised by anti-inflammatory, pro-reparative functions with increased production of cytokines such as IL-10 and promotion of tissue repair and fibrosis resolution) [40]. This indicates a mechanistic link between KCa3.1 channel activity and key inflammatory signalling cascades in immune cells.

4. Therapeutic Targeting in AF: The multifaceted involvement of KCa3.1 in AF makes it an attractive therapeutic target. An ideal AF treatment would address not only rhythm control but also the underlying atrial remodelling and KCa3.1 blockade, and KCa3.1 blockade may achieve both. A recent study by Burg et al. (PNAS Nexus 2024) provides proof of concept: they developed an allosteric inhibitor of KCa3.1 named BA6b9, and tested it in a post-myocardial infarction heart failure rat model prone to AF [42]. Chronic BA6b9 treatment prolonged the atrial effective refractory period and reduced AF inducibility, confirming the antiarrhythmic effect of SK4 inhibition. Remarkably, BA6b9 also “dramatically prevented atrial structural remodelling” in these rats. Treated animals had far less atrial fibrosis (lower collagen deposition and α-SMA expression) and reduced NLRP3 inflammasome activation in the atria compared to controls [42]. BA6b9 even reversed connexin-43 lateralisation, preserving gap junction integrity in the atria. These results illustrate that KCa3.1 blockade can simultaneously favour rhythm control and mitigate the progressive fibrosis/inflammation in AF, especially in the setting of heart failure [43]. Of note, KCa3.1 channels are preferentially expressed in atria over ventricles and are found in atrial myocytes, activated fibroblasts, and macrophages. This atrial-enriched expression provides a degree of atrial selectivity, a highly desirable feature to avoid ventricular proarrhythmia when developing anti-AF drugs [44].

## 4. KCa3.1 in Valvular Heart Disease: Fibrocalcific Remodelling of Valves

Calcific aortic valve disease (CAVD) and other valvular pathologies (e.g., rheumatic or degenerative mitral valve disease) involve chronic inflammation, fibrosis, and calcification of the valve leaflets. These processes cause valve thickening and stiffening, eventually leading to outflow obstruction (stenosis) or leakage (regurgitation). Although less studied than in vessels or myocardium, KCa3.1 appears to play analogous roles in valvular interstitial cell (VIC) activation, inflammatory cell infiltration, and osteogenic signalling in valve disease.

KCa3.1 in Valvular Interstitial Cells: KCa3.1 in Valvular Interstitial Cells: VICs are the principal effector cells in valve remodelling and exhibit marked phenotypic plasticity, transitioning from a quiescent fibroblast state to activated myofibroblast and osteoblast-like phenotypes under mechanical, inflammatory, and metabolic stimuli. Emerging evidence demonstrates that KCa3.1 channels are expressed in human aortic valve interstitial cells and are upregulated in diseased valve tissue, particularly in regions of active fibrosis and calcification.

Mechanistically, KCa3.1 functions as a regulator of sustained Ca^2+^ entry in VICs. Channel activation induces membrane hyperpolarisation, which increases the electrochemical driving force for Ca^2+^ influx through store-operated Ca^2+^ channels and related non-voltage-gated pathways. This sustained Ca^2+^ signalling is a critical trigger for the activation of downstream transcriptional programmes governing VIC proliferation, migration, and differentiation. In particular, Ca^2+^-dependent activation of ERK1/2 and NF-κB signalling pathways promotes the transition of VICs toward a myofibroblast phenotype, characterised by increased α-smooth muscle actin expression and extracellular matrix production. KCa3.1 also appears to intersect with key profibrotic pathways in VICs, including TGF-β signalling. TGF-β is a central regulator of valvular fibrosis and calcification, and its effects are partly mediated by Ca^2+^-dependent mechanisms, facilitated by KCa3.1 activity. Inhibition of KCa3.1 has been shown in fibroblast systems to attenuate TGF-β-driven myofibroblast differentiation and reduce expression of profibrotic markers, suggesting a similar mechanism is likely operative in VICs [45]. Beyond fibrosis, KCa3.1 may contribute to early osteogenic reprogramming of VICs. Sustained Ca^2+^ influx is required for activation of osteogenic transcription factors such as RUNX2, and experimental data from vascular smooth muscle and fibroblast models indicate that KCa3.1 activity supports this transition. Given the shared molecular pathways between vascular and valvular calcification, KCa3.1 is likely to facilitate coupling among inflammation, fibrosis, and calcification in valve disease [46].

Collectively, these findings position KCa3.1 as a central regulator of VIC activation, integrating Ca^2+^-dependent signalling with profibrotic and pro-osteogenic pathways. This provides a mechanistic basis for targeting KCa3.1 as a strategy to modulate early fibrocalcific remodelling in valvular heart disease

KCa3.1 and Valve Calcification: Valve calcification (especially in aortic stenosis) is an actively regulated process that involves osteoblastic differentiation of VICs. Known pro-calcific pathways (such as high phosphate, oxidised LDL, or inflammation) trigger VICs to express bone markers, including alkaline phosphatase (ALP) and the transcription factor Runx2 [47]. Notably, KCa3.1 appears to facilitate this osteogenic switch. A study on vascular calcification, which shares mechanisms with valve calcification, found that blocking KCa3.1 with TRAM-34 or using KCa3.1 shRNA significantly lowered ALP activity and Runx2 expression in calcifying VSMCs [48]. This suggests that KCa3.1 activity is required for full osteogenic signalling. By analogy, in calcific valve disease, KCa3.1 may enable the Ca^2+^ influx and signalling necessary for VICs to undergo osteoblastic differentiation. Furthermore, KCa3.1 blockade has been reported to reduce activation of pro-calcific pathways, such as NF-κB and TGF-β signalling, in a calcification model. TGF-β is a potent driver of both fibrosis and calcification in valves, promoting myofibroblast differentiation and the formation of calcific nodules in the extracellular matrix [48]. By interfering with these pathways, KCa3.1 inhibitors could mitigate the coupling of inflammation, fibrosis, and calcification in valve disease.

KCa3.1 contributes to valvular calcification through modulation of key osteogenic and inflammatory signalling pathways. Experimental data from vascular calcification models demonstrate that KCa3.1 activity enhances Ca^2+^-dependent signalling required for osteogenic differentiation, including upregulation of RUNX2, alkaline phosphatase (ALP), and osteocalcin expression. Pharmacological inhibition of KCa3.1 with TRAM-34 suppresses these osteogenic markers and reduces mineral deposition, indicating a direct role in calcification biology [49]. Mechanistically, KCa3.1-mediated membrane hyperpolarisation increases Ca^2+^ influx, which activates downstream pathways such as MAPK/ERK, PI3K/AKT, and NF-κB, all of which are known regulators of osteogenic gene transcription and inflammatory signalling. In addition, KCa3.1 has been shown to interact with TGF-β signalling, a central pathway in both fibrosis and calcification, further amplifying myofibroblast activation and extracellular matrix mineralisation [50]. These findings support a model in which KCa3.1 integrates calcium signalling, inflammation, and osteogenic transformation in valvular interstitial cells. Given the shared mechanisms between vascular and valvular calcification, targeting KCa3.1 may represent a novel strategy to interrupt early osteogenic signalling and slow the progression of calcific valve disease.

Inflammation in Valves: Calcific and fibrotic valve diseases involve infiltration of inflammatory cells (macrophages, T cells) similar to atherosclerotic lesions. These immune cells secrete proteases and osteogenic cytokines (e.g., IL-1β, IL-6, TNF-α, IFN-γ) that accelerate VIC calcification and fibrosis [51]. Their role in immune cell activation suggests they contribute to the inflammatory component of valve pathology. Infiltrating T lymphocytes and macrophages in calcified aortic valves express KCa3.1 channels (as well as Kv1.3 in T-cells) [52]. Blocking KCa3.1 may reduce pro-inflammatory cytokine production by these cells, thereby slowing valve lesion progression. Indeed, parallels can be drawn to KCa3.1’s role in atherosclerotic plaques: KCa3.1 blockade reduced macrophage and T-cell accumulation and reduced levels of inflammatory cytokines in plaques [15]. A calcified valve can be seen as an “ossified atherosclerotic plaque”; targeting KCa3.1 could similarly alleviate valve inflammation and calcification [53].

KCa3.1-dependent signalling in VIC activation and calcification: KCa3.1 likely functions as a key regulator of Ca^2+^-dependent signalling in valvular interstitial cells, as it does in fibroblasts and vascular smooth muscle cells. Channel activation induces membrane hyperpolarisation, which enhances Ca^2+^ entry through store-operated Ca^2+^ channels and sustains downstream signalling [54]. This promotes VIC proliferation and myofibroblast differentiation via the ERK1/2 and NF-κB pathways, leading to increased expression of α-SMA and extracellular matrix proteins. In parallel, KCa3.1 activity may facilitate osteogenic transition of VICs by supporting activation of RUNX2 and alkaline phosphatase, key drivers of calcific nodule formation [38]. Evidence from vascular calcification models indicates that pharmacological inhibition or silencing of KCa3.1 reduces RUNX2 expression and mineralisation, suggesting a conserved mechanism likely relevant to fibrocalcific valve disease [27]. Inflammatory signalling further amplifies this process, as cytokine-driven pathways converge on Ca^2+^-dependent transcriptional programmes regulated by KCa3.1 [55].

An overview of the major cellular sites of KCa3.1 action, dominant mechanisms, and translational implications across cardiovascular phenotypes is summarised in Table 1.

## 5. Therapeutic Implications and Future Directions

KCa3.1 Blockers as Multi-Target Cardiovascular Therapies: The convergence of evidence across IHD, AF, and valve disease positions KCa3.1 as a nexus in cardiovascular pathology. Unlike traditional therapies that often address a single facet (e.g., statins for lipids/inflammation in atherosclerosis, antiarrhythmics for electrical dysfunction in AF), KCa3.1 inhibition offers a unique multi-pronged approach. By targeting a KCa3.1 channel pivotal in vascular cells, fibroblasts, and immune cells, a single KCa3.1 blocker could simultaneously reduce vascular proliferation, fibrosis, and inflammation. This positions KCa3.1 blockade as a potential disease-modifying strategy rather than merely a symptom-control strategy.

Several KCa3.1 inhibitors have been developed and tested in preclinical or early clinical settings. The antifungal agent clotrimazole blocks KCa3.1 at nM concentrations but is unsuitable for chronic human use due to off-target effects on cytochrome P450 [57]. TRAM-34, an analogue of clotrimazole, is widely used experimentally and has shown efficacy in animal models without notable toxicity at effective physiological doses [17]. The selective KCa3.1 blocker senicapoc (ICA-17043) was developed for sickle cell disease and reached Phase III trials [56]. Senicapoc demonstrated good safety and tolerability in humans (Phases I–III), with dose-dependent mild adverse effects, including diarrhoea, but an overall very favourable safety profile. Although senicapoc did not ultimately reduce vaso-occlusive crises in sickle cell disease, its ability to inhibit KCa3.1 in patients was confirmed, and it improved blood haemoglobin concentrations and significantly reduced red cell dehydration, thereby validating target engagement [17,56]. The repurposing of senicapoc for cardiovascular indications is a promising possibility, particularly given its oral availability and safety record.

Another avenue is the design of allosteric inhibitors (such as the BA6b9 mentioned above) that may achieve higher selectivity or organ-targeted effects [52]. The success of BA6b9 in a rat AF/HF model suggests that medicinal chemistry efforts to optimise KCa3.1 blockers could yield next-generation therapeutics for AF, particularly for patients with concomitant heart failure or structural heart disease [42]. These could complement existing antiarrhythmic drugs and potentially reduce the need for invasive procedures by maintaining sinus rhythm through reverse remodelling [58].

Despite the promise, some challenges and considerations remain. One concern is the potential off-target effects and the physiological roles of KCa3.1 in other tissues. KCa3.1 is generally not expressed in most excitable tissues (e.g., brain, skeletal muscle), which is why blockers have a relatively low risk of acute toxicity. However, evidence indicates that KCa3.1 underlies the slow afterhyperpolarisation in certain neurons and that blocking it could affect neuronal excitability or sensory function [44]. Another consideration is the immune-modulating effect: while dampening inflammation is beneficial in chronic cardiovascular disease, one must ensure that long-term KCa3.1 inhibition does not predispose patients to infection or impair immune surveillance [59]. Encouragingly, animal studies have shown that moderate KCa3.1 blockade does not grossly immunosuppress (mice on TRAM-34 handled viral infection normally), likely because KCa3.1 is just one of multiple channels involved in immune cell activation [42].

Biomarkers and Patient Selection: As KCa3.1-based therapies move toward clinical consideration, identifying which patient groups might benefit most is important. For instance, patients with refractory AF and extensive atrial fibrosis (e.g., persistent AF with heart failure) may benefit significantly from a KCa3.1 blocker to reverse fibrosis and reduce AF burden [40]. Likewise, post-MI patients or those with stents could potentially receive a KCa3.1 inhibitor to prevent restenosis and atherosclerosis progression. In valvular disease, early-phase trials might target patients with moderate calcific aortic stenosis to see if KCa3.1 blockade slows the increase in valve gradient over time [41]. Noninvasive imaging of inflammation (e.g., PET scans of macrophage activity in plaques or valves) could serve as biomarkers to assess drug efficacy.

KCa3.1 also has potential as a biomarker of disease activity and remodelling. Increased KCa3.1 expression has been demonstrated in atherosclerotic plaques, atrial tissue in AF, and fibrotic cardiac models, suggesting that it reflects active inflammatory and fibroproliferative signalling. In this context, KCa3.1 may serve as a marker of disease activity rather than a static measure of disease burden. Circulating or tissue-derived measures of KCa3.1 expression, including transcriptomic profiling or protein detection in peripheral blood mononuclear cells, could serve as surrogates for immune activation and remodelling status. In AF, KCa3.1 expression may correlate with the burden of atrial fibrosis and arrhythmia persistence, offering potential utility for risk stratification and prediction of treatment response, including ablation outcomes [4,44]. Similarly, in atherosclerosis and post-PCI settings, KCa3.1-related signalling may identify patients at higher risk of restenosis or plaque progression [60].

Combination Therapies: KCa3.1 blockers may synergise with other treatments. For example, combining a KCa3.1 blocker with a statin or a PCSK9 inhibitor may yield additive protection in coronary disease, with the former targeting cellular proliferation/inflammation and the latter addressing lipid-driven mechanisms [27]. In AF, a KCa3.1 blocker could be combined with ablative therapy or conventional antiarrhythmics to stabilise rhythm and prevent recurrence by remodelling the substrate [61].

Combination strategies may be particularly attractive because KCa3.1 inhibition targets Ca^2+^-dependent immune activation and fibroproliferative remodelling, which are not fully addressed by standard therapies [62]. In coronary disease, KCa3.1 blockade could be combined with lipid-lowering therapy to provide complementary mechanisms: statins and PCSK9 inhibitors reduce lipid-driven risk, whereas KCa3.1 inhibition suppresses vascular smooth muscle cell phenotypic switching and immune-mediated plaque inflammation [63,64]. In post-PCI settings, KCa3.1 inhibition could also be explored alongside contemporary anti-proliferative drug-eluting stents or anti-inflammatory approaches to reduce neointimal hyperplasia and inflammatory restenosis [10]. In AF, combining KCa3.1 inhibition with rhythm-control strategies, such as catheter ablation or antiarrhythmic drugs, may reduce recurrence by attenuating fibroinflammatory substrate progression, and combinations with upstream therapies, such as renin–angiotensin system blockade, may be synergistic given their shared effects on atrial fibrosis signalling [40]. In valvular disease, a rational future direction is to evaluate anti-calcific and anti-inflammatory strategies alongside evaluation, with the aim of interrupting early osteogenic signalling and cytokine-driven VIC activation [47].

Gene-silencing strategies targeting KCa3.1 represent an emerging therapeutic avenue with potential for high specificity. Preclinical studies using siRNA and shRNA approaches have demonstrated that suppression of KCa3.1 expression attenuates fibroblast activation, reduces vascular smooth muscle cell proliferation, and limits inflammatory cytokine production. These approaches directly reduce channel expression rather than modulating channel activity, offering a complementary mechanism to pharmacological inhibition [32]. Advances in RNA-based therapeutics and targeted delivery systems, including nanoparticle-mediated and tissue-specific delivery, may enable selective modulation of KCa3.1 in cardiovascular tissues while minimising systemic effects [65]. Although currently limited to experimental models, gene-silencing approaches provide proof of concept for precise modulation of KCa3.1-driven pathways and represent a promising direction for future translational research.

**Ongoing and future research**

Future work should focus on translating preclinical insights into clinically actionable strategies, with emphasis on human validation, target engagement, and early-phase therapeutic evaluation.

Translational studies are needed to validate KCa3.1 expression and function in human cardiovascular tissues, including atrial myocardium, atherosclerotic plaques, and calcified valves, and to correlate these with disease severity and clinical phenotype [66].

Early-phase clinical trials of KCa3.1 inhibitors should assess safety, target engagement, and preliminary efficacy using clinically relevant endpoints such as AF burden, restenosis rates, and progression of valvular stenosis.

The development of more selective or tissue-targeted KCa3.1 modulators is an important priority to enhance therapeutic efficacy while minimising off-target effects [11].

Mechanistic studies should further define upstream regulators of KCa3.1 expression, including neurohormonal and inflammatory pathways, and clarify interactions with established cardiovascular therapies such as renin–angiotensin system inhibitors and anti-inflammatory agents [67].

Integration of biomarker strategies, including molecular profiling and inflammation-targeted imaging, will also be important for identifying patients most likely to benefit from KCa3.1-directed therapies and for monitoring treatment response. These approaches will be critical to define the clinical utility of KCa3.1 as both a therapeutic target and a biomarker across cardiovascular disease phenotypes.

## 6. Conclusions

KCa3.1 channels, encoded by the KCA3.1 gene, have emerged as important molecular orchestrators in cardiovascular disease. They operate at the crossroads of electrical excitability, fibrotic remodelling, and immune activation, processes fundamental to IHD, AF, and valvular diseases. In ischaemic cardiovascular disease, KCa3.1 contributes to vascular smooth muscle proliferation and chronic inflammation, whereas its role in endothelial cells is primarily linked to the regulation of Ca^2+^-dependent signalling and vasodilatory function [68]. In the atria, KCa3.1 contributes to arrhythmogenic electrical remodelling and fosters the fibrosis and inflammation that underpin AF. In the valves, KCa3.1 likely mirrors these pathogenic roles by supporting the transformation of quiescent valve interstitial cells into pro-fibrotic, calcifying cells, in concert with infiltrating inflammatory cells.

The consistent theme across these conditions is that KCa3.1 amplifies pathologic Ca^2+^-dependent signalling in multiple cell types, whether it’s a proliferating VSMC, an activated fibroblast, or an inflammatory T-cell. By doing so, it links cellular excitation to long-term structural changes (hyperplasia, fibrosis, calcification) and functional impairment in the cardiovascular system. This makes KCa3.1 an especially compelling therapeutic target: blocking this single channel can produce multifaceted benefits, as demonstrated in various preclinical models, from reducing atherosclerotic plaque burden to suppressing AF and fibrosis in failing hearts.

The path ahead involves translating these insights into clinical therapies. Fortunately, the groundwork has been laid by the development of KCa3.1 inhibitors in other fields (immunology, haematology), providing tools and safety data to build upon. Future studies will determine the optimal use of KCa3.1 blockade in patients, whether as a standalone treatment or in combination, and which disease stage yields the greatest benefit. What is now clear is that KCa3.1 is a central mediator of cardiovascular pathophysiology, and its modulation holds promise to transform our approach to diseases such as atherosclerosis, AF, and aortic stenosis.

Future evidence addressing current knowledge gaps, such as the role of KCa3.1 modulation in preventing vein graft disease or stent restenosis, will provide pivotal information to aid the utilisation of currently available and tested inhibitors to treat such conditions, which so far have no widely accepted interventions, thus improving health outcomes for a large group of patients.

## Figures and Tables

**Table 1:** KCa3.1 in cardiovascular disease. Key mechanisms and translational opportunities.

Condition	Where KCa3.1 Acts	Core Mechanism	Dominant Remodelling Axis	What Blockade Achieves in Models	Translational Angle
Ischaemic heart [10,11,15,16] disease and atherosclerosis	Endothelium; vascular smooth muscle cells; macrophages; T cells	Sustains Ca^2+^ signalling in vascular and immune cells, while in endothelial cells KCa3.1 contributes to Ca^2+^ dependent signalling that supports endothelium dependent hyperpolarisation and nitric oxide mediated vasodilation [9]	Vascular remodelling and inflammation	Reduced plaque burden; reduced neointima; fewer inflammatory infiltrates	Anti-remodelling adjunct for CAD; post-PCI restenosis prevention
Atrial fibrillation [36]	Atrial myocytes; fibroblasts; macrophages	Shortens atrial repolarisation; promotes fibroblast activation and cytokine-driven substrate progression	Electrical and fibroinflammatory remodelling	Prolonged ERP; reduced AF inducibility; reduced atrial fibrosis and inflammation	Disease-modifying rhythm control; atrial-selective target
Valvular heart disease [51,53]	Valvular interstitial cells; infiltrating immune cells	Supports myofibroblast activation and osteogenic signalling with inflammatory amplification	Fibrocalcific remodelling	Reduced myofibroblast activity; reduced calcific nodule formation	Medical therapy concept to slow AS progression
Therapeutic targeting [17,42,52,56]	System level	Selective KCa3.1 inhibition with repurposing potential	Multi-compartment remodelling control	Attenuation of remodelling, inflammation, and arrhythmia burden	Oral candidates exist; clear path to early-phase trials

AF, atrial fibrillation. AS, aortic stenosis. CAD, coronary artery disease. ERP, effective refractory period. PCI, percutaneous coronary intervention.

## Data Availability

No new data were created or analysed in this study.
